# Changes in glycosylated proteins in colostrum and mature milk and their implication

**DOI:** 10.3389/fnut.2023.1161310

**Published:** 2023-06-16

**Authors:** Jing Lu, Wenyuan Zhang, Changlu Ma, Xiaoyang Pang, Ying Dai, Tong Zhu, Jinqi Liu, Lina Xing, Shuwen Zhang, Jiaping Lv

**Affiliations:** ^1^Beijing Advanced Innovation Center for Food Nutrition and Human Health, Beijing Engineering and Technology Research Center of Food Additives, School of Food and Health, Beijing Technology and Business University, Beijing, China; ^2^Institute of Food Science and Technology, Chinese Academy of Agricultural Science, Beijing, China; ^3^Department of Food and Bio-Engineering, Beijing Vocational College of Agriculture, Beijing, China

**Keywords:** glycoproteins, colostrum, mature milk, host defense, IgA

## Abstract

**Introduction:**

Glycosylation is one of the essential post-translational modifications that influences the function of milk proteins.

**Methods:**

In the present study, 998 proteins and 764 glycosylated sites from 402 glycoproteins were identified in human milk by TMT labeling proteomics. Compared to human milk proteins, the glycoproteins were mainly enriched in cell adhesion, proteolysis, and defense/immune process.

**Results:**

The abundance of 353 glycosylated sites and their 179 parent proteins was quantified. After normalization to their parent protein’s abundance, 78 glycosylated sites in 56 glycoproteins and 10 glycosylated sites in 10 glycoproteins were significantly higher in colostrum and mature milk, respectively. These changed glycoproteins were mainly related to host defense. Intriguingly, one glycosylated site (Asp144) in IgA and two glycosylated sites (Asp38 and Asp1079) in tenascin are significantly upregulated even though their protein abundance was downregulated during lactation.

**Discussion:**

This study helps us figure out the critical glycosylated sites in proteins that might influence their biological function in an unbiased way.

## 1. Introduction

Human milk is critical for the development and health of newborns. It contains various nutrients with different biological functions. Proteins are the fourth most abundant component in it. They provide essential amino acids and peptides for the growth of newborns. Meanwhile, milk proteins also play roles in the maturation of the infant immune system, digestion of other milk components, and protection against infection (1). The content of milk proteins changes during lactation, and individual proteins changes differently. These changes were thought to be adapted to the needs of newborns. Though paramount efforts have been made to understand the importance and changes of milk proteins during lactation, the occurrence and changes of glycosylated proteins in human milk and their biological consequence remained elusive.

Glycosylation refers to the covalent bond of oligosaccharides to proteins. Glycosylation influences various aspects of protein, including the structure, stability, trafficking, recognition, and biological function (2). The functions of glycosylated proteins have been extensively studied in disease. The abnormal changes in protein glycosylation were related to the progression of cancer (3). Another essential aspect of glycosylation is that glycosylation of proteins could modulate the immune system and the onset of inflammation response (4). Though glycosylated proteins and their biological functions were extensively investigated in other biological samples, this information remained largely elusive in human milk. Efforts have been made to understand how milk proteins were glycosylated and their potential implications. Typically, glycosylation helps stabilize the structure of human milk proteins. It also influences the antibacterial activity of lactotransferrins (5). The glycans attached to milk proteins could be utilized by bifidobacterial (6), reshaping the composition of newborns’ microbiota (7). The glycosylation changed dynamically during lactation (8–11). However, most of the studies focused on the changes in glycosylation sites and did not count the parent protein abundance when investigating the changes during lactation. These changes could be attributed to changes in either the protein abundance or the glycosylation degree. Thus, previous findings might be hard to interpret if they were solely based on glycosylated sites without normalization to the parent protein abundance.

In the present study, proteomics and glycosylomics were simultaneously applied to both human colostrum and mature milk. We jointly combined the outputs from proteomics and glycoproteomics to investigate the changes in protein glycosites and abundance during lactation. After normalizing to protein abundance, we found 88 glycosylation sites significantly changed during lactation. GO analysis suggested that these differential glycosites were mainly from immune-related glycoproteins. Collectively, these results helped researchers to comprehensively understand the changes in proteins’ glycosylation during lactation and their potential consequences.

## 2. Materials and methods

### 2.1. Sample collection

Human colostrum was collected from 10 healthy mothers aged between 25–40 years old at day 1–7 postpartum. Mature milk was collected from 10 healthy mothers aged between 25–38 years old at 4–6 months of lactation. The foremilk samples were collected from right breast of participants at 10:00–11:00 am by using breast pump. The colostrum and mature milk samples were randomly pooled to 3 tubes (3,3, and 4 samples per tube, 10 mL per sample resulting 30 mL, 30 mL and 40 mL in each tube) respectively and stored at −80°C for further analysis. All the participants were mastitis-free and with no other diseases. Written informed consents were obtained from all participants. The patients/participants provided their written informed consent to participate in this study. The studies involving human participants were reviewed and approved by Institute of Food Science and Technology, Chinese Academy of Agricultural Science, Beijing, China (CAASIFST2015003).

### 2.2. Protein digestion

Milk samples were centrifuged at 1500 g for 10 min at 10°C to remove the fat layer. The protein concentrations in milk were determined by the bicinchoninic acid (BCA) assay (12). The dithiothreitol was added to the sample to a final concentration of 5 mM for 30 min at 56°C. Afterwards, iodoacetamide was added to a final concentration of 11 mM and incubated for 15 min at room temperature in the dark. And the urea concentration of the sample was diluted to less than 2 M. Trypsin was added at a mass ratio of 1:50 (trypsin:protein) and digested overnight at 37°C. Then, trypsin was added again at a mass ratio of 1:100 (trypsin:protein) and the digestion was continued for 4 h.

### 2.3. TMT labeling

Digested peptides were desalted with Strata X C18 (Phenomenex) and freeze-dried. In brief, the pH of the 100 μL samples were adjusted to 3 using 10 μL of 1% TFA solution. The Strata X columns were wetted once with 1 mL of 100% acetonitrile and then rinsed three times with 1 mL of 0.1% TFA. The samples were loaded onto the columns by centrifugation at 800 rpm for 3 min. After loading, the columns were washed three times with 1 mL of 0.1% TFA. To elute the peptides, 100 μL of elution buffer (60% acetonitrile and 0.1% TFA) was applied to the columns and centrifuged at 800 rpm for 3 min. The eluted peptides were then freeze-dried. Peptides were solubilized with 0.5 M triethylamonium bicarbonat and labeled according to the instruction of TMT kit (Thermofisher). Briefly, the labeling reagent was thawed and dissolved in acetonitrile, mixed with peptides and incubated at room temperature for 2 h. The labeled peptides were mixed, desalted and freeze-dried (label used: TMT-126 for colostrum, TMT-131 for mature milk).

### 2.4. HPLC fractionation

The tryptic peptides were fractionated by high pH reverse-phase HPLC using Agilent 300 Extend C18 (5 μm particles, 4.6 mm ID, 250 mm length). Briefly, peptides were first separated with a gradient of 8 to 32% acetonitrile (pH 9.0) over 60 min into 60 fractions. Then, the peptides were combined into 10 fractions (for human milk proteomics) and 4 fractions (for human milk glycolproteomics) and freeze dried.

### 2.5. Glycopeptides enrichment

Each of 4 peptides-fractions (for human milk glycolproteomics) was dissolved in 40 μL enrichment buffer solution (80% acetonitrile/1% trifluoroacetic acid), and transferred to a hydrophilic microcolumn (HILIC column, Dalian Institute of Chemistry Physics, Chinese Academy of Sciences, China) (13), and the enrichment was completed by centrifugation at 4000 g for 15 min. The microcolumns were then washed 3 times with enrichment buffer. The glycopeptides were then eluted with 10% acetonitrile and lyophilized. Then, the lyophilized glycopeptides were reconstituted in 50 μL of 50 mM ammonium bicarbonate buffer dissolved in heavy oxygen water (H_2_^18^O), 2 μL PNGase F glycosidase (New England Biolabs, United Kingdom) was added, and the peptides were digested overnight at 37°C. Finally, the salt was removed according to the instructions of C18 ZipTips (Sigma, United States), and the samples were freeze-dried for LC/MS analysis.

### 2.6. LC-MS/MS analysis

The trypsin digested and enriched glycopeptides were dissolved in 0.1% formic acid and 2% acetonitrile (solvent A), and separated by EASY-nLC 1,000 ultra-high performance liquid chromatography, respectively. Solvent B contained 0.1% formic acid and 90% acetonitrile. The elution gradient was as follows: 0–42 min, 4%–20% B; 42–54 min, 20%–35% B; 54–57 min, 35%–80% B; 57–60 min, 80% B, the flow rate was 350 nL/min.

The UPLC separated peptides/glycopeptides were subjected to NSI source followed by tandem mass spectrometry (MS/MS) in FusionTM (Thermo Scientific, United States). The electrospray voltage applied was 2.0 kV. The precursor and fragment ions were detected and analyzed by Orbitrap. The *m*/*z* scan range was 350 to 1,550 for full scan at a resolution of 60,000. The start of MS/MS scan was set as 100 *m*/*z*, the resolution of Orbitrap scanning was 15,000. The data aquation mode was set as DDA, the top 20 precursor ions were sequentially transferred to HCD collision cell, 35% collision energy was applied for fragmentation and the fragmented ions were subsequently analyzed. In order to improve the utilization of the mass spectrum, the automatic gain control (AGC) was set to 5E4, the signal threshold was set to 5,000 ions/s, the maximum injection time was set to 200 ms, and the dynamic exclusion time of the tandem mass spectrometry scan was set to 15 s to avoid repeated scanning of the parent ions.

### 2.7. Data analysis

The resulting MS/MS data were processed using Maxquant search engine (v.1.5.2.8). Tandem mass spectra were searched against human UniProt database concatenated with reverse decoy database. Trypsin/P was specified as cleavage enzyme allowing up to 2 missing cleavages. The mass tolerance for precursor ions was set as 20 ppm in first search and 5 ppm in main search, and the mass tolerance for fragment ions was set as 0.02 Da. Carbamidomethyl on Cys was specified as fixed modification and acetylation modification and oxidation on met were specified as variable modifications. For glycopeptides analysis, the deamidation of asparagine to aspartic acid with incorporation of ^18^O was set as variable modification as well. Then quantification method was set to TMT 6-plex. The “re-quantify” option was applied in MaxQuant analysis. The FDR for protein identification and PSM identification was adjusted to <1%.

### 2.8. Bioinformatic analysis

The Gene Ontology (GO) and KEGG pathway enrichment was analyzed by using DAVID Bioinformatics Resources 6.8^1^ (14). The protein classification was analyzed by Panther^2^ (15).

## 3. Results

### 3.1. Identification of glycosylated/unglycosylated proteins in human milk

**Figure 1:**
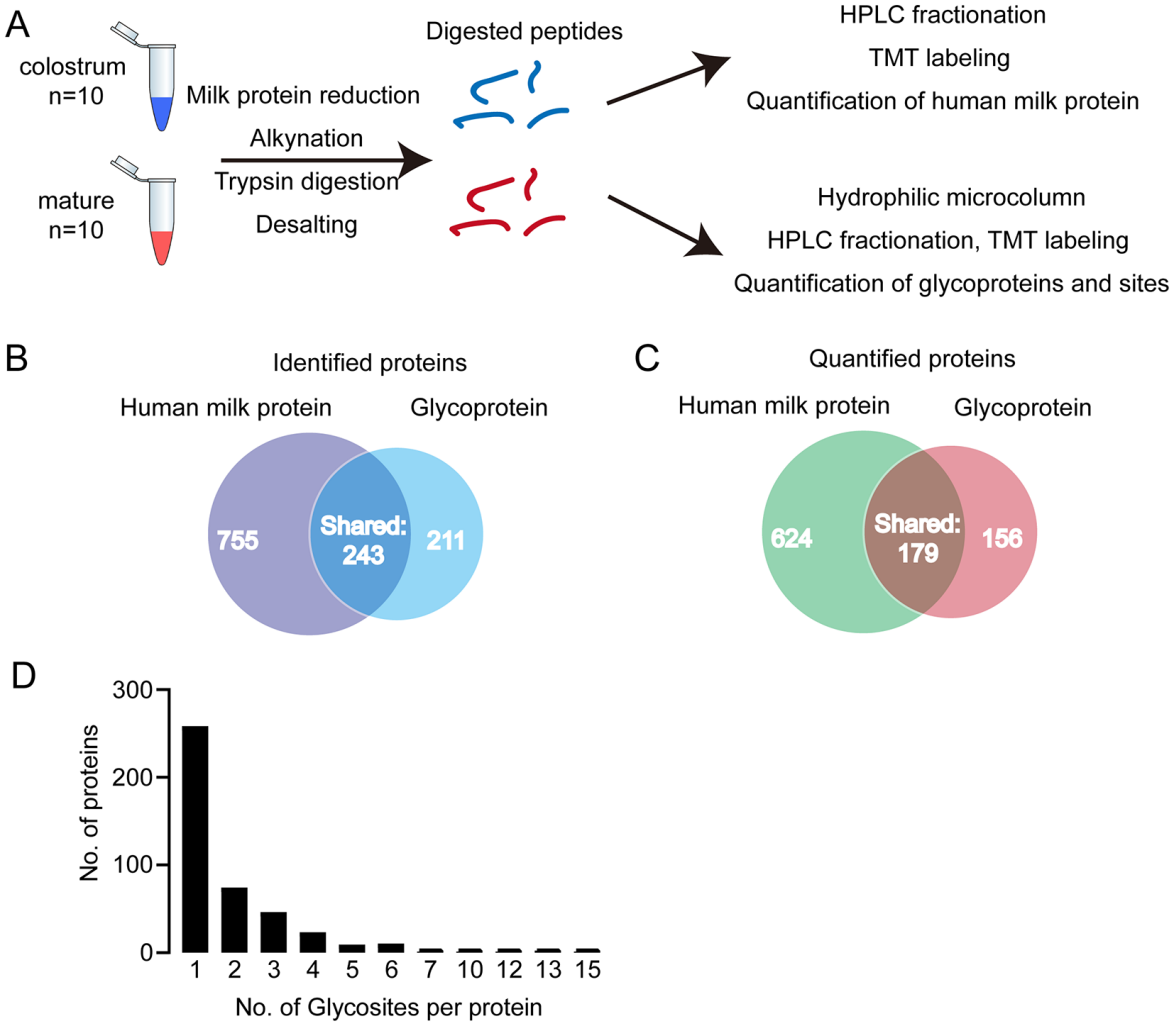
**(A)** Analytical approach of present study; **(B)** Venn diagram of identified and quantified human milk (HM) proteins and glycoproteins; **(C)** distribution of glycosylated sites per protein. **(D)** Distribution of singly and multiply glycosylated proteins.

In present study, we first analyzed the proteins in colostrum and mature milk by proteomics. In total, 998 human milk proteins were identified and 803 of them could be quantified. Then, the glycopeptides were enriched by HILIC column for glycoproteomics (Figure 1A). In total, 402 glycoproteins were identified, 335 of them could be quantified (Figures 1B,C). And 234 out of identified glycoproteins were present in human milk proteomics analysis. In other words, 168 glycoproteins were not found in human milk proteomics analysis. We speculated that the abundance of these 168 glycoproteins was too low to be detected in the raw milk. These 168 glycoproteins were enriched by HILIC columns, thereby enabling them to be detected in glycoproteomics analysis. Collectively, 1,166 proteins including glycoproteins and human milk proteins, i.e., 764 + 234 + 168 = 1,166 were found in present study, and 34.5% (402/1166) of them were glycosylated. And 32% of the identified glycosylated proteins contained one glycosylation site (Figure 1D). Five proteins were highly glycosylated with more than 10 glycosites. The highly glycosylated proteins were as follows: tenascin contained 15 glycosylated sites; polymeric immunoglobulin receptor contained 13 glycosylated sites; 12 glycosylated sites were observed in lactotransferrin and attractin; 10 glycosylated sites were identified in mucin-4. Compared to previously reported glycosylated sites, six new glycosylated sites were identified in lactotransferrin (Supplementary Table S1), including Asn 71, Asn 489，Asn 557, Asn 572, Asn 576, and Asn 663 (MS/MS spectrum of these sites were shown in Supplementary Figure S1). In addition, we also observed glycosylated sites of major proteins in human milk, including Asn 69 and Asn 74 of α-S1-casein, Asn 33 of κ-casein, and Asn 90 of α-lactalbumin. To our knowledge, there were no records of κ-casein glycosylated sites in previously reports (Supplementary Table S1 and Supplementary Figure S1).

### 3.2. Gene enrichment analysis of glycosylated proteins

**Figure 2:**
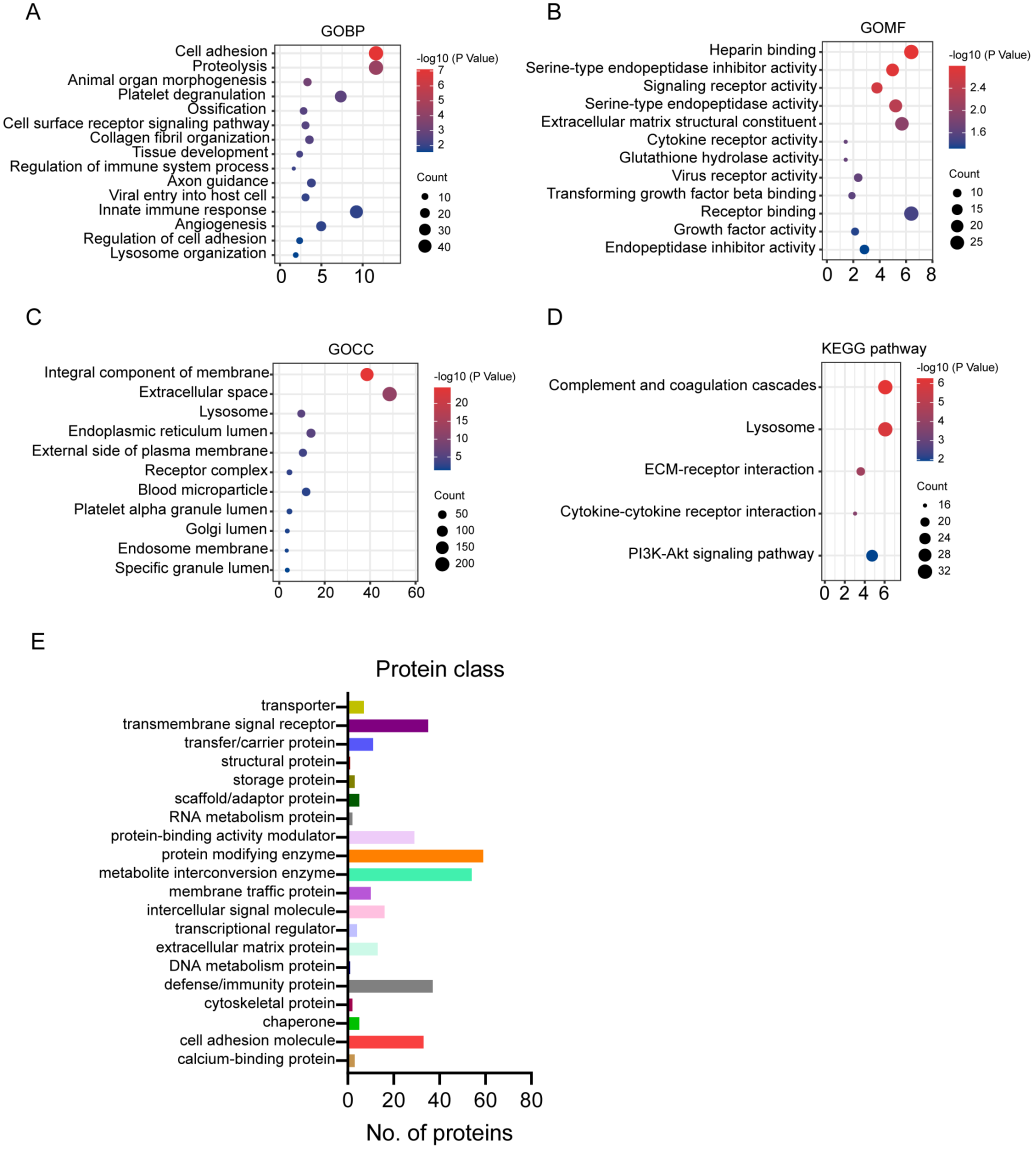
Gene Ontology (GO), KEGG pathway enrichment of glycosylated proteins to human milk proteins **(A–D)**: **(A)** GO biological process; **(B)** GO molecular function; **(C)** GO cellular component; **(D)** KEGG pathway; protein classification of glycosylated proteins in human milk **(E)**.

Compared to human milk proteome, the glycosylated proteins were enriched in biological process related to cell adhesion, proteolysis, organelle development, platelet degranulation, and immune response (Figure 2A). And the molecular function of glycosylated milk proteins was mainly binding, protease activity, and receptor activity (Figure 2B). Most of the glycosylated proteins were from membrane and extracellular space (Figure 2C). They were involved in the pathways of complement and coagulation cascade, lysosome, and extra cellular matrix (ECM) -receptor interaction (Figure 2D).

The glycosylated proteins identified in the present study were further classified according to the protein function category in Panther (see footnote 2) (15, 16). According to the classification, 113 glycoproteins were enzymes, including 59 protein modifying enzymes and 54 metabolite interconversion enzymes (Figure 2E). And 51 out of the 59 protein modifying enzymes are protease. And 19 out of 54 metabolite interconversion enzymes were related to carbohydrate metabolism. Furthermore, 37 glycosylated proteins were defense/immunity proteins. Transmembrane signal receptor and cell adhesion molecules also counted for more than 30 proteins (Figure 2E).

### 3.3. Significantly changed glycosylated proteins during lactation

In present study, 292 glycosylated sites in 173 proteins significantly changed between colostrum and mature milk under cutoffs: fold change >1.5, *p* < 0.05. As discussed in the introduction section, the 292 differential glycosylated sites could be due to their protein abundance changes. After normalization by their protein abundance, the intensity of 78 glycosylated sites in 56 proteins were significantly higher in colostrum, while the intensity of 10 glycosylated sites in 10 proteins were significantly higher in mature milk (Table 1). Thirty-six glycosylated sites in nineteen host defense related proteins significantly changed during lactation, 30 of which were significantly higher in colostrum, including five (Asn 71, Asn 521, Asn 534, Asn 557, and Asn 576) out of 13 glycosylated sites in lactotransferrin, one (Asn 499) out of 13 glycosylated sites in Polymeric immunoglobulin receptor, two (Asn 220 and Asn 321) out of 3 glycosylated sites in CD36, Asn 205 of two glycosylated sites in Ig alpha-2 chain C region, Asn 176 in Ig gamma-2 chain C region, Asn 46 out of six glycosylated sites in Ig mu chain C region, and seven (Asn 184, Asn 327, Asn 1,034, Asn 1,093, Asn 1,119, Asn 1,366, and Asn 1,485) out of 15 glycosylated sites in tenascin. While, six glycosylated sites in four host defense related proteins were significantly higher in mature milk, including Asn 144 out of two glycosylated sites in Ig alpha-1 chain C region, Asn 106 and Asn 271 in alpha-1-antichymotrypsin, Asn 38 and Asn 1,079 out of 15 glycosylates sites in tenascin, and Asn 103 out of 3 glycosylated sites in alpha-1-acid glycoprotein 1. Twenty-seven glycosylated sites in ten cell-adhesion-related proteins significantly changed during lactation (Table 1). Among them, 20 glycosylated sites were significantly higher in colostrum, while only 3 of them were significantly higher in mature milk, including two glycosylated sites in tenascin mentioned above, and Asn 352 out of six identified glycosylated sites in olfactomedin-4. And fourteen glycosylated sites in 13 enzymes were significantly higher in colostrum, including 4 protease, 2 sulfatase, and 7 other enzymes. One of the two glycosylated sites (Asn 69) in α-S1-casein was significantly higher in colostrum.

**Table 1:** Significantly changed glycosylated sites and proteins between colostrum and mature milk (1.5 fold change, *p* < 0.05).

UniProt number	Glycosylated Asn position	Name	Gene name	Protein quantification (colostrum/mature)	Sites quantification (colostrum/mature)	Normalized site quantification (colostrum/mature)	Function
O00300	165	Tumor necrosis factor receptor superfamily member 11B	TNFRSF11B	1.01 ± 0.15	2.62 ± 0.28	2.61 ± 0.12	Apoptosis
O14786	150	Neuropilin-1	NRP1	2.85 ± 0.04	5.31 ± 0.09	1.86 ± 0.05	Angiogenesis; neurogenesis
O14786	521	Neuropilin-1	NRP1	2.85 ± 0.04	5.06 ± 0.46	1.78 ± 0.19	
O75882	383	Attractin	ATRN	2.23 ± 0.01	3.73 ± 0.06	1.67 ± 0.02	Host defense
P00738	241	Haptoglobin	HP	1.91 ± 0.11	2.98 ± 0.11	1.56 ± 0.03	Host defense
P01008	128	Antithrombin-III	SERPINC1	1.03 ± 0.02	0.5 ± 0.03	0.48 ± 0.02	Blood coagulation
P01011	106	Alpha-1-antichymotrypsin	SERPINA3	2.61 ± 0.24	1.54 ± 0	0.59 ± 0.05	Host defense
P01011	271	Alpha-1-antichymotrypsin	SERPINA3	2.61 ± 0.24	1.34 ± 0.01	0.51 ± 0.04	
P01033	101	Metalloproteinase inhibitor 1	TIMP1	0.66 ± 0.04	1.36 ± 0.15	2.07 ± 0.12	Protease inhibitor
P01042	205	Kininogen-1	KNG1	0.82 ± 0	1.68 ± 0.08	2.04 ± 0.09	Blood coagulation; host defense
P01133	404	Pro-epidermal growth factor	EGF	1.05 ± 0.02	1.84 ± 0.15	1.76 ± 0.11	Growth factor
P01833	499	Polymeric immunoglobulin receptor	PIGR	2.4 ± 0	6.29 ± 0.12	2.61 ± 0.05	Host defense
P01859	176	Ig gamma-2 chain C region	IGHG2	0.79 ± 0.03	1.3 ± 0.06	1.65 ± 0.13	Host defense
P01871	46	Ig mu chain C region	IGHM	3.51 ± 0.04	5.85 ± 0.06	1.67 ± 0.04	Host defense
P01876	144	Ig alpha-1 chain C region	IGHA1	2.93 ± 0.03	1.37 ± 0.45	0.47 ± 0.16	Host defense
P01877	205	Ig alpha-2 chain C region	IGHA2	2.5 ± 0.07	5 ± 0.08	2 ± 0.03	Host defense
P02749	253	Beta-2-glycoprotein 1	APOH	0.89 ± 0.03	1.77 ± 0.04	1.98 ± 0.1	Blood coagulation
P02749	162	Beta-2-glycoprotein 1	APOH	0.89 ± 0.03	1.48 ± 0.04	1.66 ± 0	
P02751	542	Fibronectin	FN1	0.6 ± 0.03	1.02 ± 0.08	1.7 ± 0.04	Host defense; cell adhesion
P02763	103	Alpha-1-acid glycoprotein 1	ORM1	2.52 ± 0.37	0.63 ± 0	0.25 ± 0.04	Host defense
P02788	534	Lactotransferrin	LTF	1.01 ± 0	1.95 ± 0.25	1.92 ± 0.24	Host defense; transport
P02788	71	Lactotransferrin	LTF	1.01 ± 0	1.75 ± 0.15	1.73 ± 0.15	
P02788	576	Lactotransferrin	LTF	1.01 ± 0	1.7 ± 0.14	1.68 ± 0.14	
P02788	521	Lactotransferrin	LTF	1.01 ± 0	1.63 ± 0.02	1.61 ± 0.02	
P02788	557	Lactotransferrin	LTF	1.01 ± 0	1.55 ± 0.03	1.54 ± 0.03	
P02794	112	Ferritin heavy chain	FTH1	1 ± 0.03	1.95 ± 0.14	1.94 ± 0.08	Iron storage
P04004	242	Vitronectin	VTN	0.84 ± 0.02	1.31 ± 0.02	1.56 ± 0.07	Cell adhesion
P04114	1,523	Apolipoprotein B-100	APOB	0.4 ± 0	1.89 ± 0.03	4.73 ± 0.03	Lipid transport
P04278	396	Sex hormone-binding globulin	SHBG	3.17 ± 0.86	1.74 ± 0.59	0.54 ± 0.04	Lipid binding
P05164	483	Myeloperoxidase	MPO	1.59 ± 0.49	3.88 ± 0.79	2.47 ± 0.27	Host defense
P05164	355	Myeloperoxidase	MPO	1.59 ± 0.49	3.3 ± 0.47	2.13 ± 0.37	
P07602	101	Prosaposin	PSAP	1.14 ± 0.03	1.96 ± 0.02	1.72 ± 0.06	Lipid metabolism
P07602	426	Prosaposin	PSAP	1.14 ± 0.03	1.8 ± 0.05	1.58 ± 0.08	
P08174	95	Complement decay-accelerating factor	CD55	3.21 ± 0.23	5.56 ± 0.3	1.73 ± 0.03	Host defense
P10909	103	Clusterin	CLU	3.45 ± 0.04	6.05 ± 0.06	1.75 ± 0	Host defense, apoptosis
P11717	400	Cation-independent mannose-6-phosphate receptor	IGF2R	1.52 ± 0.15	0.9 ± 0.02	0.59 ± 0.07	Transport
P12273	105	Prolactin-inducible protein	PIP	12.81 ± 0.54	25.4 ± 3.11	1.98 ± 0.16	Acting binding
P12821	695	Angiotensin-converting enzyme	ACE	2.27 ± 0.17	3.63 ± 0.04	1.61 ± 0.14	Blood pressure
P15586	279	N-acetylglucosamine-6-sulfatase	GNS	0.64 ± 0.04	1.07 ± 0.11	1.67 ± 0.07	Glycosaminoglycan/keratan sulfate catabolic process
P16671	321	Platelet glycoprotein 4	CD36	3 ± 0.22	5.69 ± 0.17	1.89 ± 0.08	Cell adhesion; transport; host defense
P16671	220	Platelet glycoprotein 4	CD36	3 ± 0.22	5.23 ± 0	1.75 ± 0.13	
P19440	120	Gamma-glutamyltranspeptidase 1	GGT1	3.3 ± 0.17	7.31 ± 1.8	2.21 ± 0.43	Glutathione biosynthesis
P23284	148	Peptidyl-prolyl cis-trans isomerase B	PPIB	0.76 ± 0.01	2.11 ± 0.02	2.76 ± 0.01	Protein folding
P24821	184	Tenascin	TNC	8.03 ± 0.21	44.43 ± 8.03	5.54 ± 0.84	Cell adhesion; host defense
P24821	1,119	Tenascin	TNC	8.03 ± 0.21	32.2 ± 9.31	4.01 ± 1.07	
P24821	1,093	Tenascin	TNC	8.03 ± 0.21	36.38 ± 3.75	4.51 ± 0.36	
P24821	327	Tenascin	TNC	8.03 ± 0.21	34.03 ± 1.56	4.26 ± 0.33	
P24821	1,034	Tenascin	TNC	8.03 ± 0.21	34.73 ± 5.62	4.35 ± 0.84	
P24821	166	Tenascin	TNC	8.03 ± 0.21	33.73 ± 7.09	4.24 ± 1	
P24821	1,485	Tenascin	TNC	8.03 ± 0.21	24.75 ± 0.43	3.07 ± 0.14	
P24821	1,366	Tenascin	TNC	8.03 ± 0.21	17.37 ± 2.99	2.16 ± 0.42	
P24821	38	Tenascin	TNC	8.03 ± 0.21	3.74 ± 0.16	0.47 ± 0.03	
P24821	1,079	Tenascin	TNC	8.03 ± 0.21	2.19 ± 0.27	0.27 ± 0.03	
P25311	127	Zinc-alpha-2-glycoprotein	AZGP1	1.13 ± 0.01	5.65 ± 1.01	5.01 ± 0.97	Lipid degradation; cell adhesion
P25311	128	Zinc-alpha-2-glycoprotein	AZGP1	1.13 ± 0.01	5.54 ± 0.27	4.9 ± 0.31	
P25311	109	Zinc-alpha-2-glycoprotein	AZGP1	1.13 ± 0.01	2.05 ± 0.05	1.81 ± 0.07	
P25311	112	Zinc-alpha-2-glycoprotein	AZGP1	1.13 ± 0.01	1.93 ± 0.02	1.71 ± 0	
P47710	69	Alpha-S1-casein	CSN1S1	1.96 ± 0.08	5.09 ± 0.03	2.6 ± 0.12	Milk protein
P48723	184	Heat shock 70 kDa protein 13	HSPA13	1.41 ± 0.06	2.3 ± 0.01	1.63 ± 0.07	Protein refolding
P49368	308	T-complex protein 1 subunit gamma	CCT3	0.62 ± 0.01	1.42 ± 0.01	2.3 ± 0.06	Protein folding
P50897	197	Palmitoyl-protein thioesterase 1	PPT1	1.05 ± 0.19	2.28 ± 0.24	2.23 ± 0.62	Lysosomal degradation
P51993	91	“Alpha-(1,3)-fucosyltransferase 6”	FUT6	1.55 ± 0.06	2.96 ± 0.09	1.92 ± 0.14	Glycosylation
P53634	53	Dipeptidyl peptidase 1	CTSC	2 ± 0.02	3.1 ± 0.1	1.54 ± 0.04	Protease
P98160	89	Basement membrane-specific heparan sulfate proteoglycan core protein	HSPG2	4.5 ± 1.01	10.8 ± 0.95	2.49 ± 0.78	Angiogenesis
Q02487	392	Desmocollin-2	DSC2	2.27 ± 0.2	5.33 ± 0.1	2.36 ± 0.25	Cell adhesion
Q06481	541	Amyloid-like protein 2	APLP2	1.52 ± 0.07	2.5 ± 0.06	1.64 ± 0.12	Cell signalling
Q08380	551	Galectin-3-binding protein	LGALS3BP	4.05 ± 0.18	6.42 ± 0.18	1.59 ± 0.03	Cell adhesion; host defense
Q14515	169	SPARC-like protein 1	SPARCL1	3.37 ± 0.17	1.48 ± 0.11	0.44 ± 0.06	Calcium binding
Q24JP5	280	Transmembrane protein 132A	TMEM132A	1.47 ± 0.12	2.33 ± 0.07	1.59 ± 0.09	
Q6UX06	136	Olfactomedin-4	OLFM4	6.96 ± 1.66	19.49 ± 0.32	2.86 ± 0.64	Cell adhesion
Q6UX06	193	Olfactomedin-4	OLFM4	6.96 ± 1.66	13.44 ± 0.99	1.97 ± 0.32	
Q6UX06	352	Olfactomedin-4	OLFM4	6.96 ± 1.66	2.88 ± 0.01	0.43 ± 0.1	
Q6W4X9	2,366	Mucin-6	MUC6	1.65 ± 0.35	22.77 ± 4.01	14.39 ± 5.54	Mucosa component
Q6WN34	114	Chordin-like protein 2	CHRDL2	1.18 ± 0.02	2.03 ± 0.09	1.71 ± 0.11	Differentiation; chondrogenesis; osteogenesis
Q7Z7M0	1,201	Multiple epidermal growth factor-like domains protein 8	MEGF8	3.61 ± 0.07	6.44 ± 0.19	1.79 ± 0.09	Organ morphogenesis
Q7Z7M0	1990	Multiple epidermal growth factor-like domains protein 8	MEGF8	3.61 ± 0.07	1.78 ± 0.35	0.49 ± 0.09	
Q8IWU5	171	Extracellular sulfatase sulf-2	SULF2	1.73 ± 0.2	3.66 ± 0.1	2.13 ± 0.19	Organ morphogenesis
Q8NBJ4	108	Golgi membrane protein 1	GOLM1	1.77 ± 0.07	3.07 ± 0.01	1.74 ± 0.08	Host defense
Q8NHP8	88	Putative phospholipase B-like 2	PLBD2	1.31 ± 0.21	2.15 ± 0.45	1.63 ± 0.08	Lipid metabolism
Q8WXI7	12,414	Mucin-16	MUC16	1.42 ± 0.26	10.96 ± 0.14	7.87 ± 1.53	Host defense; mucosa component; cell adhesion
Q92484	238	Acid sphingomyelinase-like phosphodiesterase 3a	SMPDL3A	0.96 ± 0.01	1.5 ± 0.02	1.56 ± 0.04	Nucleoside triphosphate catabolic
Q99102	2049	Mucin-4	MUC4	3.19 ± 0.28	5.64 ± 0.46	1.78 ± 0.3	Cell adhesion; mucosa component
Q99102	1809	Mucin-4	MUC4	3.19 ± 0.28	5.18 ± 0.11	1.63 ± 0.11	
Q99102	1802	Mucin-4	MUC4	3.19 ± 0.28	5.15 ± 0.43	1.61 ± 0.01	
Q9BYF1	103	Angiotensin-converting enzyme 2	ACE2	1.87 ± 0.01	3.1 ± 0.26	1.66 ± 0.13	Blood pressure
Q9H173	193	Nucleotide exchange factor SIL1	SIL1	1.05 ± 0.04	2.04 ± 0.11	1.95 ± 0.19	Protein transport
Q9HC84	4,960	Mucin-5B	MUC5B	1.31 ± 0.58	5.86 ± 2.61	5.44 ± 4.4	Mucosa component
Q9Y2D9	305	Zinc finger protein 652	ZNF652	0.67 ± 0.03	1.54 ± 0.21	2.29 ± 0.21	Transcription
Q9Y646	179	Carboxypeptidase Q	CPQ	0.82 ± 0.07	1.56 ± 0.16	1.91 ± 0.04	Proteolysis
Q9Y6R7	1,063	IgGFc-binding protein	FCGBP	2.11 ± 0.13	3.76 ± 0.07	1.79 ± 0.14	Mucosa component

## 4. Discussion

The number of identified glycoproteins and glycosylated sites was relatively large, and new glycosylated sites were identified compared to previously reports (Supplementary Table S1). This result implied the robustness of the glycoproteomics technique used in this study. Notably, in our study, the changes of proteins glycosylated sites were normalized to their abundance, enabling us to correctly interpret the glycosylate sites alternations during lactation in an unbiased way.

Enzymes, defense/immune proteins, and cell adhesion proteins constitute the major part in human milk glycoproteins (Figure 2). This observation was in agreement with the main biological function classification of glycoproteins observed in other biological samples (17).

### 4.1. Changes of host defense glycoproteins

Among the changed glycosylated proteins, host defense proteins accounted for the largest proportion (Table 1). This indicated that the glycosylation of host defense proteins might play important role in proteins’ properties during lactation. Immunoglobulins were critical for the protection function of human milk to infants and they are known for their glycosylation (18). The dynamic changes in glycosylation of immunoglobulins, especially of immunoglobulin A1 and A2 during lactation were investigated by several groups 10, 11, 18. In agreement with these studies, three out of four glycosylated sites in immunoglobulins (IgG, IgM, and IgA2) significantly decreased during lactation, except for Asn 144 in IgA1 (Table 1). We observed that the glycosylation of Asn 144 in IgA was upregulated even its protein abundance decreased as the lactation stage prolonged. Certain types of glycans attached to Asn 144/205 of IgA increased during lactation (10). Both protein and glycosylation level of IgA decreased from day 1 to day 30 in human milk by using coomassie- or pro-Q-stained electrophoretic methods (19). However, the decrease of glycosylation level is significantly less than that of protein abundance, which also indicated the upregulation of glycosylation of IgA during lactation. Glycosylation has been shown to affect bacterial and pathogen attachment and clearance based on the structural characterization of IgA in milk (20). We hypothesize that the elevation of Asn144 on IgA may help the immunoglobulin maintain its protective effect in infants which compensated for the gradual loss of protein abundance during lactation. This assumption needs further investigation.

Another host defense glycoprotein that gained our attention is lactotransferrin. The glycosylation of lactotransferrin and its change during lactation has been investigated by several groups (10, 19, 21). The glycosylation of lactotransferrin could influence its iron binding activity (22), proteolysis (23) and anti-adhesive properties of pathogens (21). The three well-studied glycosylated sites (Asn156, 497, and 642) of lactotransferrin were all identified in the present study. Furthermore, six novel glycosylated sites of lactotransferrin (Asn 71, 489, 557, 572, 576, and 663) were identified in the present study. This could be due to the improvement of the analysis techniques and the different samples analyzed among studies. It was reported that the total glycosylation level of lactotransferrin decreased as the lactation prolonged (21). We also observed the decrease of Asn 71, 521, 534, 557,576 during lactation. The significantly higher intensity of Asn 534 was observed in colostrum compared to mature milk 8, whereas another study only identified this site in mature milk (11). Similarly, Wang et al. (11) only identified Asn 521 of lactotransferrin in mature milk. The discrepancies could be due to the different time points that mature milk was collected among studies. For example, in our study, the mature milk was obtained from 4–6 months postpartum and in the study of Wang et al. (11), the mature milk was collected from 15 days to 3 months. Thus, in our opinion, it is better to collect the milk samples per month or even per week during lactation to figure out the law of glycosylation changes.

In addition to the abovementioned immunoglobulins and lactotransferrin, we have also identified glycosylated sites in host defense proteins such as haptoglobin and clusterin. To our knowledge, the effects of glycosylation on these host defense proteins in milk were not well investigated. Due to the critical function of human milk, especially colostrum to newborns’ protection, the understanding of glycosylation on protein’s function might help to deepen our understanding of human milk. Furthermore, the specific glycosylated protein in colostrum might provide us clues for medical therapies.

### 4.2. Changes of glycosylated sites in enzymes

Though large number of glycosylated enzymes identified and quantified in the present study, only 14 glycosylated sites in 13 enzymes significantly changed during lactation. This indicated that the glycosylation of the enzymes was relatively stable during lactation. The influences of glycosylation on enzymes were mainly related to protein folding, secretion, stability, binding activity, structure, and their enzymatic activity (24). These influences could be positive or negative, depending on the nature of glycosylated sites (24). One (Asn120) out of 3 glycosylated sites in γ-glutamyltranspeptidase 1 (GGT1) was significantly higher in colostrum. This glycosylated site has also been identified by other reports (8, 25). However, they did not observe significantly change of this site during lactation. Since 1970s, the activity of GGT1 was measured in breast milk (26), its activity was the highest in colostrum (12,613 U/L at 1 week postpartum) and decreased to 501 U/L at 6 month. The abundance of GGT1 was 3.3 times higher in colostrum than that in mature milk in the present study (Table 1). However, the effect of glycosylation on GGT1 was not well investigated yet. We also searched the studies related to the other 12 changed glycosylated enzymes. Unfortunately, little was known of their presence and precise function in human milk, letting alone the role of glycosylation.

### 4.3. Changes of glycosylates sites in cell adhesion proteins

The glycans on cells’ surface helped cells to interact with the other cells (17). Cell adhesion is essential for assembling single cells into three-dimensional organization of tissues and organs (27). There is increasing evidence showing that the changes in the N-glycan structure of these adhesive molecules affect cell–cell and cell-extracellular matrix interactions, thereby affecting cell adhesion and migration (28). To our knowledge, the function of cell adhesion related proteins in human milk was not investigated. In the present study, 27 glycosylated sites in 10 cell adhesion related proteins were significantly changed during lactation (Table 1). Among the proteins involved in cell adhesion, tenascin is the interesting one. It is first described as a cell adhesion modulator (29, 30). However, in human milk, it is famous for its human immunodeficiency virus (HIV)-1–neutralizing properties (31). In the present study, 15 glycosylation sites were identified on tenascin, and 10 sites changed differently between colostrum and mature milk. Among them, 8 sites were higher in colostrum, whereas 2 sites were higher in mature milk. In consistency, Cao et al. (8, 9) also observed the higher amount of Asn (166, 1,034, 1,093, 1,119, 1,366, and 1,485) in colostrum. Though the abundance of tenascin decreased during lactation (Table 1) (19, 32), the abundance of Asn 38 and Asn 1,039 decreased less than its parent protein, leading the upregulation of these two glycosylated sites (Table 1). It has been reported that the glycosylation of tenascin influences the proliferation of marine neuron stem cell (33). And another study indicated that the glycosylation of tenascin affected its binding capacity and structure, protecting it from proteolysis (34). Thus, these 10 glycosylated sites might influence the structure and function of tenascin in colostrum and mature milk, which needs to be further investigated.

Besides the proteins discussed above, other changed glycosylated sites in various proteins were observed in the present study (Supplementary Table S1). Due to the critical biological function of colostrum on the protection and development of infants, the glycosylation status of colostrum and its changes during lactation need to be comprehensively investigated.

Due to the challenges of collecting human milk samples, a limited number of milk samples were used in this study, which were obtained from lactating mothers of varying ages. These limitations are acknowledged, in our future work, we will make efforts to increase the sample size and reduce the variation of lactation mothers. This work is our first attempt to understand the glycosylation of milk proteins during lactation.

## 5. Conclusion

In sum, we normalized the changes of glycosylated sites during lactation to their corresponding parental proteins abundance. This provided a valuable resource for studying the glycosylation of protein in colostrum and its changes during lactation. The changed glycosylated proteins in colostrum and mature human milk are mainly involved in the function of host defense. Due to the critical biological function of colostrum on protection and development of infants, our results here help to improve our understanding of glycosylated proteins in colostrum and design better food for health of infants. Furthermore, the detail study of exact modification of physiologically important proteins in milk also help produce vaccination and drugs for disease treatment, such as the possible use of tenascin in HIV fending.

## Data availability statement

The original contributions presented in the study are publicly available. This data can be found here: http://proteomecentral.proteomexchange.org/cgi/GetDataset?ID=PXD039934.

## Ethics statement

The studies involving human participants were reviewed and approved by Institute of Food Science and Technology, Chinese Academy of Agricultural Science, Beijing, China. The patients/participants provided their written informed consent to participate in this study.

## Author contributions

JL and JPL contributed to conception and design of the study. JL and WZ contributed to sample analysis. JL, CM, XP, and SZ contributed to data analysis. YD, TZ, JQL, and LX contributed to sample collection. JL, WZ, and JPL contributed to draft writing and revision. JL and JPL contributed to funding acquisition. All authors contributed to the article and approved the submitted version.

## Funding

This work was supported by National Natural Science Foundation of China (32272355 and 31671878).

## Conflict of interest

The authors declare that the research was conducted in the absence of any commercial or financial relationships that could be construed as a potential conflict of interest.

## Publisher’s note

All claims expressed in this article are solely those of the authors and do not necessarily represent those of their affiliated organizations, or those of the publisher, the editors and the reviewers. Any product that may be evaluated in this article, or claim that may be made by its manufacturer, is not guaranteed or endorsed by the publisher.

## Footnotes

^1^ http://david.ncifcrf.gov/

^2^ http://pantherdb.org/
