# Supplementary material for: Changes in glycosylated proteins in colostrum and mature milk and their implication
Source: Front Nutr. 2023 Jun 16;10:1161310. doi: 10.3389/fnut.2023.1161310 (PMC10311556; doi:10.3389/fnut.2023.1161310)
Supplement: Supplementary file 2 [file Data_Sheet_1.pdf]

Raw file	Scan	Method	Score	m/z	Gene names
2_3	15177	FTMS; HCD	104.95	616.65	LTF N71

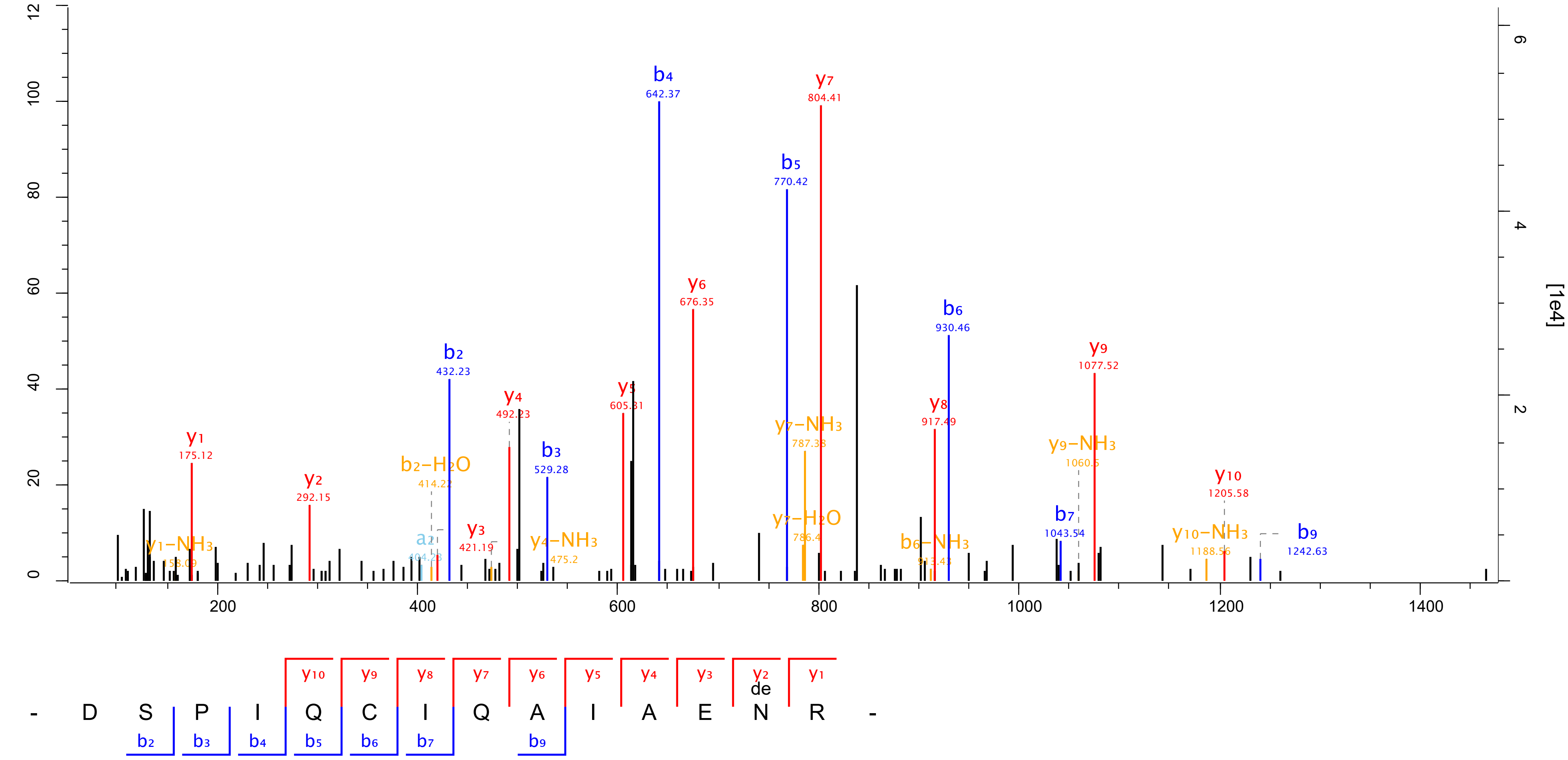

Figure S1, MS/MS spectrum of new identified N-glycosylated sites in lactotransferrin (LTF) and κ-casein (CSN3)

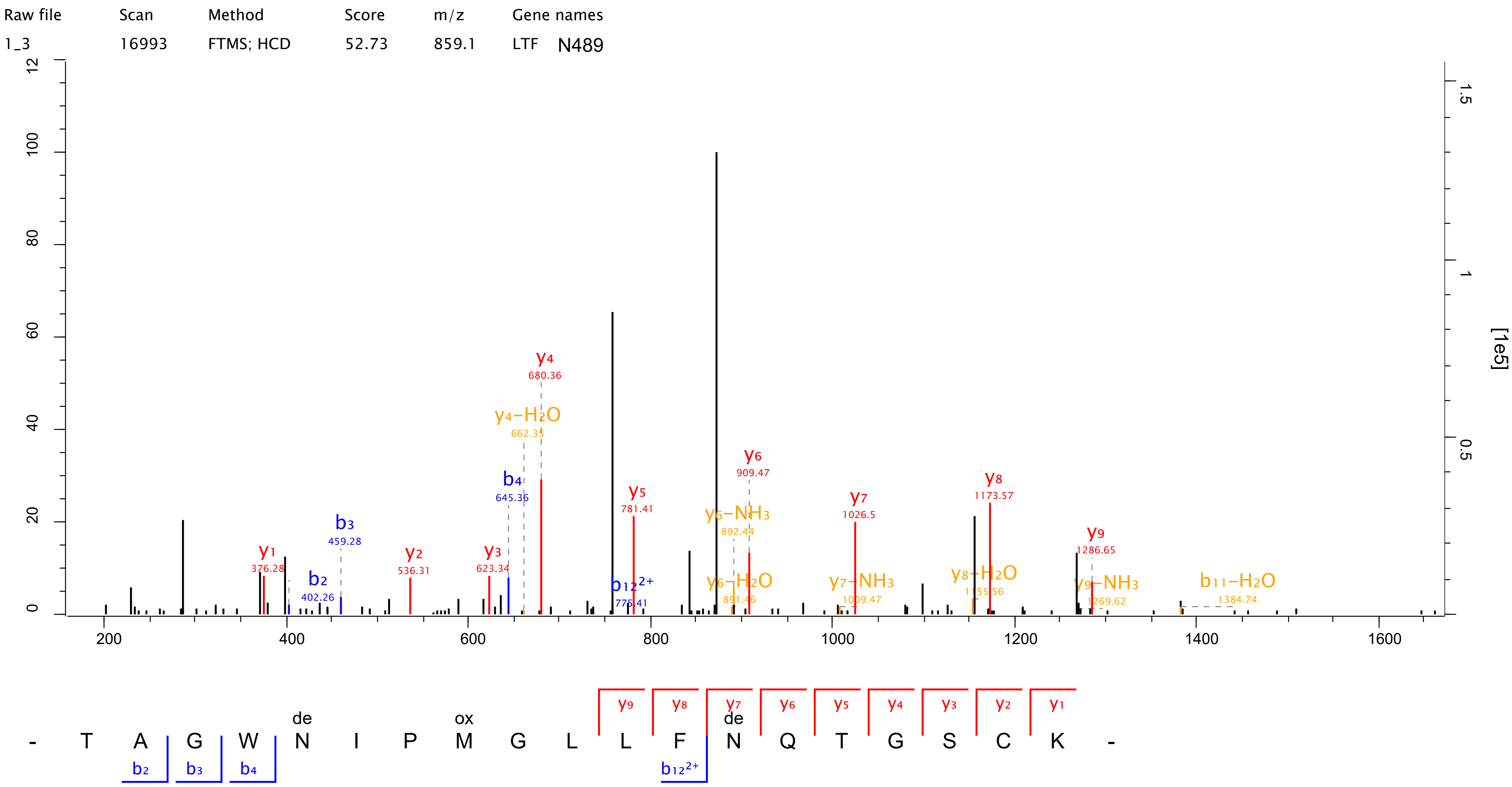

Figure S1, MS/MS spectrum of new identified N-glycosylated sites in lactotransferrin (LTF) and κ-casein (CSN3)

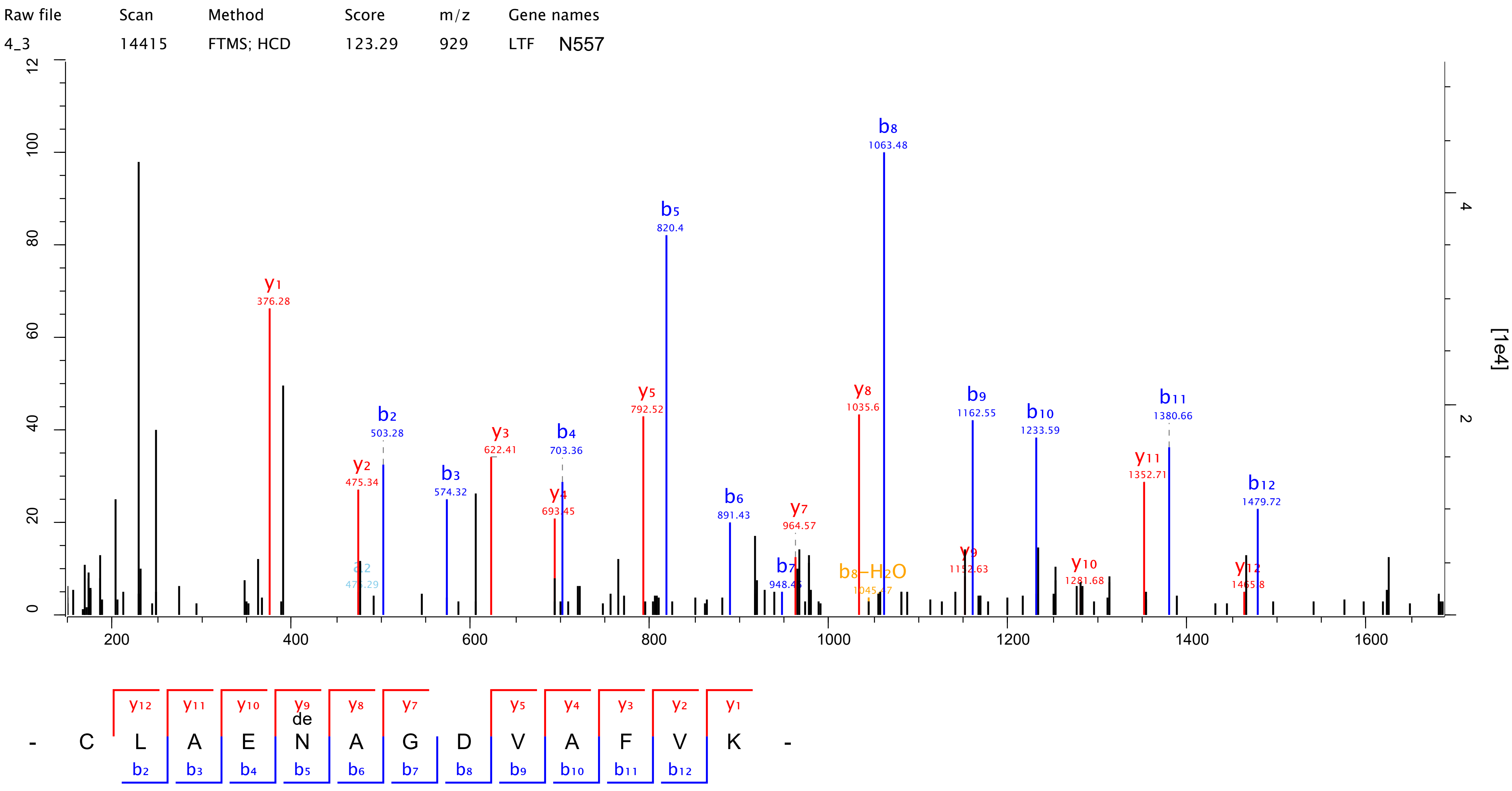

Figure S1, MS/MS spectrum of new identified N-glycosylated sites in lactotransferrin (LTF) and κ-casein (CSN3)

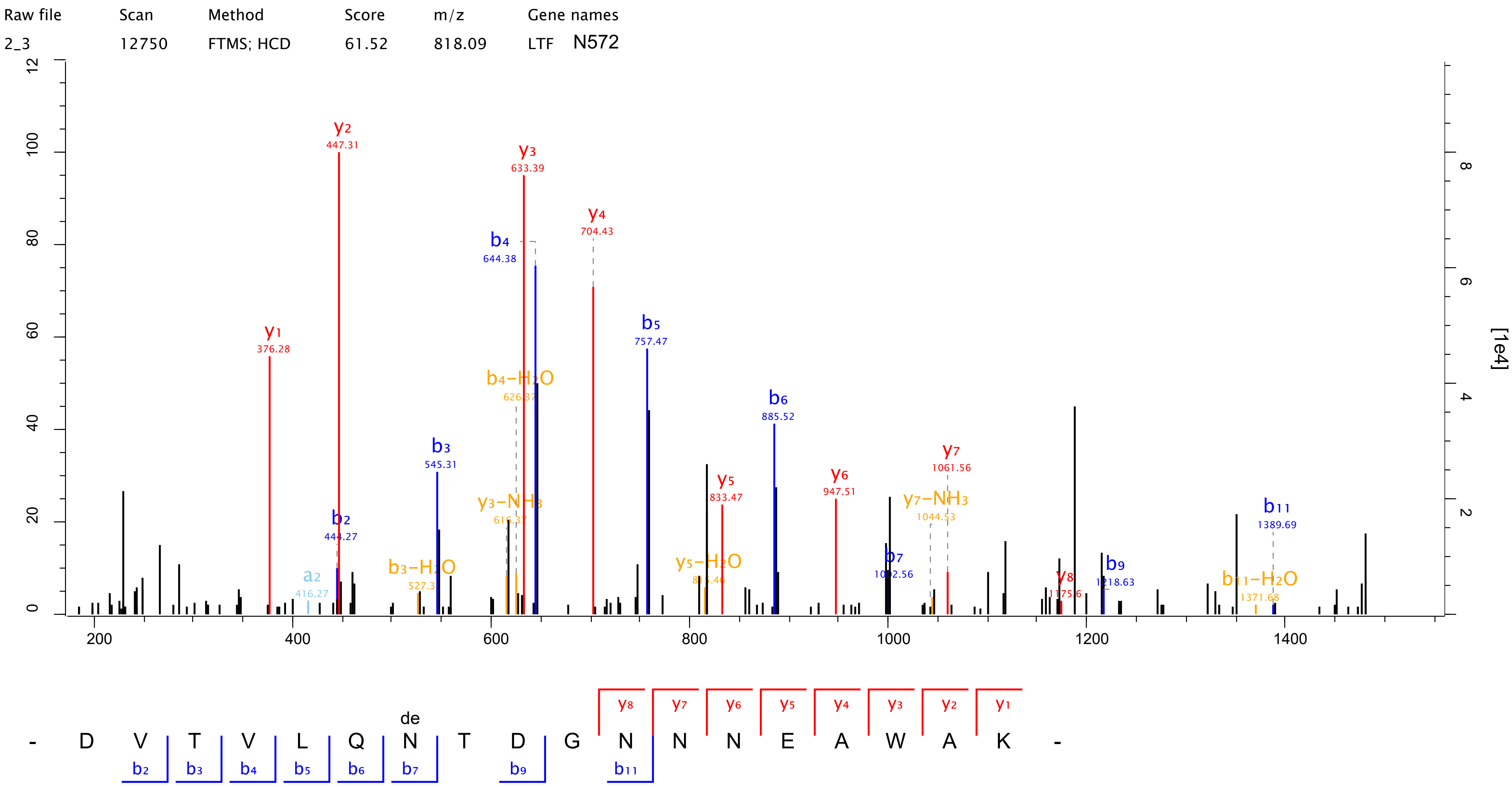

Figure S1, MS/MS spectrum of new identified N-glycosylated sites in lactotransferrin (LTF) and κ-casein (CSN3)

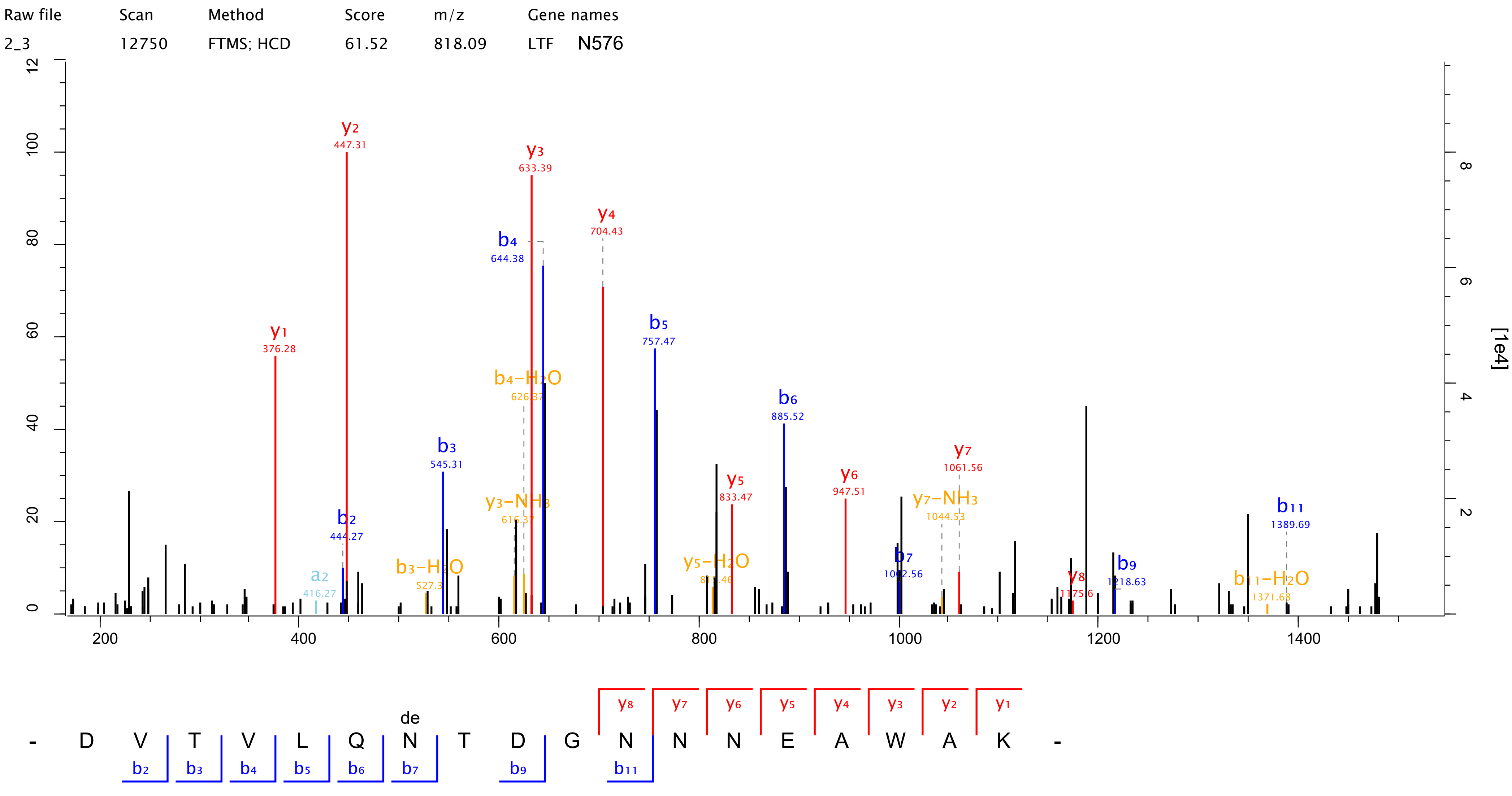

Figure S1, MS/MS spectrum of new identified N-glycosylated sites in lactotransferrin (LTF) and κ-casein (CSN3)

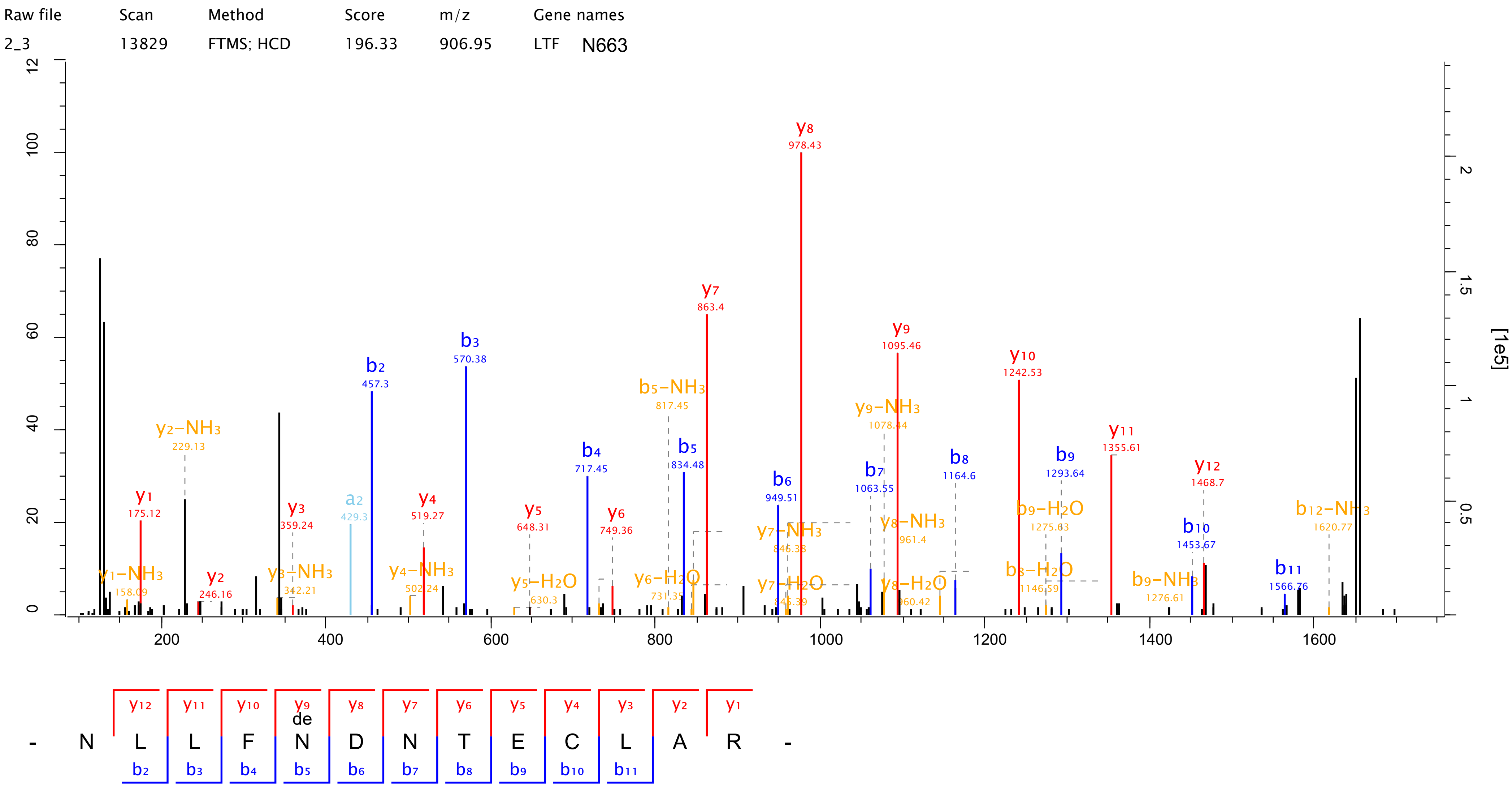

Figure S1, MS/MS spectrum of new identified N-glycosylated sites in lactotransferrin (LTF) and κ-casein (CSN3)

Raw file	Scan	Method	Score	m/z	Gene names
4_3	8212	FTMS; HCD	61.41	595.8	CSN3 N33

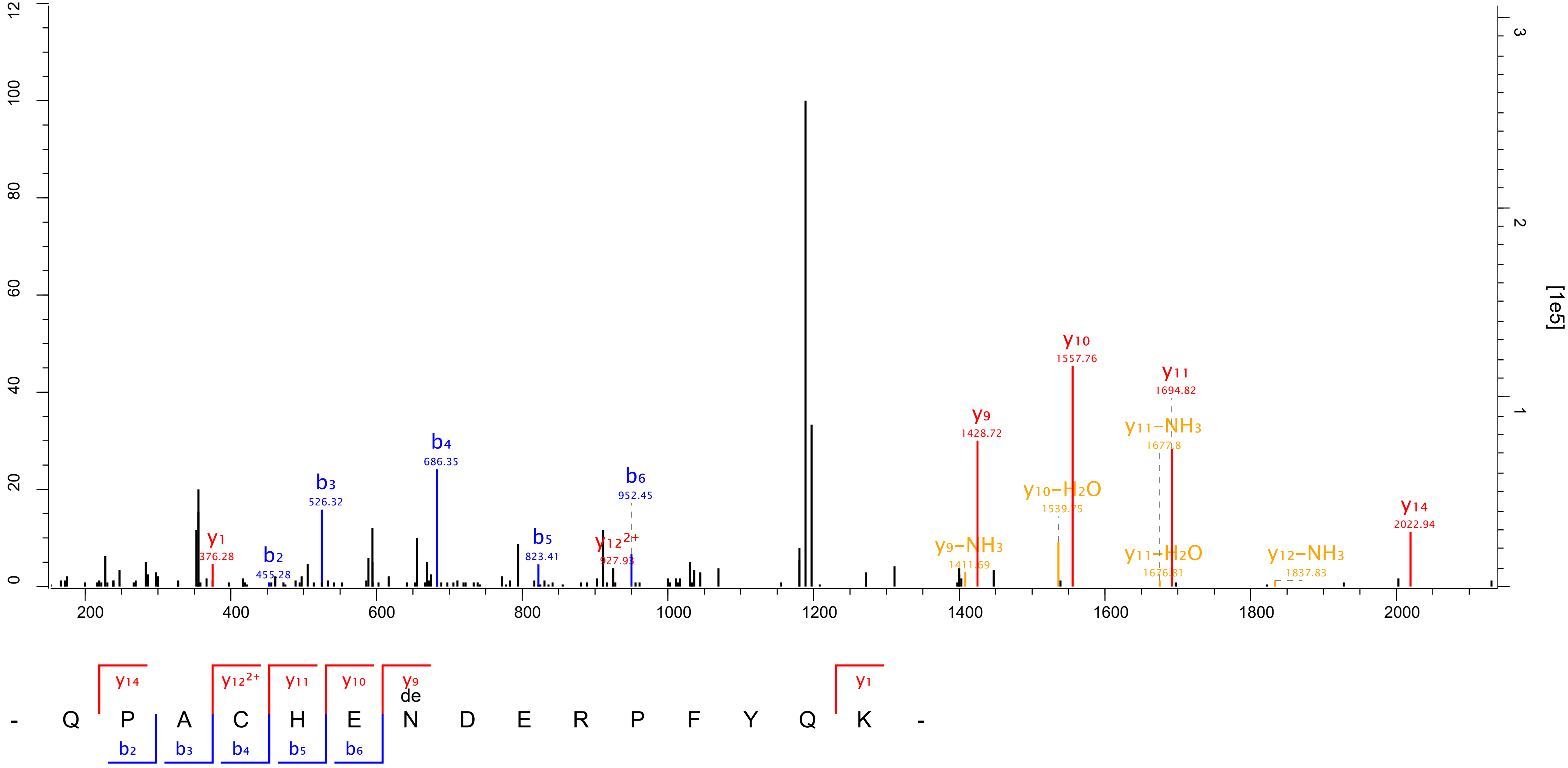

Figure S1, MS/MS spectrum of new identified N-glycosylated sites in lactotransferrin (LTF) and κ-casein (CSN3)
